# Intestinal microbiota-derived tryptophan metabolites are predictive of Ah receptor activity

**DOI:** 10.1080/19490976.2020.1788899

**Published:** 2020-08-12

**Authors:** Fangcong Dong, Fuhua Hao, Iain A. Murray, Philip B. Smith, Imhoi Koo, Alyssa M. Tindall, Penny M. Kris-Etherton, Krishne Gowda, Shantu G. Amin, Andrew D. Patterson, Gary H. Perdew

**Affiliations:** a Department of Veterinary and Biomedical Sciences and the Center for Molecular Toxicology and Carcinogenesis, The Pennsylvania State University, University Park, PA, USA; b The Huck Institutes of the Life Sciences, the Pennsylvania State University, University Park, PA, USA; c Department of Nutritional Sciences, The Pennsylvania State University, University Park, PA, USA; d Department of Pharmacology, Penn State College of Medicine, Hershey, PA, USA

**Keywords:** Ah receptor, tryptophan, indole, metabolome, stool, cecal content

## Abstract

Commensal microbiota-dependent tryptophan catabolism within the gastrointestinal tract is known to exert profound effects upon host physiology, including the maintenance of epithelial barrier and immune function. A number of abundant microbiota-derived tryptophan metabolites exhibit activation potential for the aryl hydrocarbon receptor (AHR). Gene expression facilitated by AHR activation through the presence of dietary or microbiota-generated metabolites can influence gastrointestinal homeostasis and confer protection from intestinal challenges. Utilizing untargeted mass spectrometry-based metabolomics profiling, combined with AHR activity screening assays, we identify four previously unrecognized tryptophan metabolites, present in mouse cecal contents and human stool, with the capacity to activate AHR. Using GC/MS and LC/MS platforms, quantification of these novel AHR activators, along with previously established AHR-activating tryptophan metabolites, was achieved, providing a relative order of abundance. Using physiologically relevant concentrations and quantitative gene expression analyses, the relative efficacy of these tryptophan metabolites with regard to mouse or human AHR activation potential is examined. These data reveal indole, 2-oxindole, indole-3-acetic acid and kynurenic acid as the dominant AHR activators in mouse cecal contents and human stool from participants on a controlled diet. Here we provide the first documentation of the relative abundance and AHR activation potential of a panel of microbiota-derived tryptophan metabolites. Furthermore, these data reveal the human AHR to be more sensitive, at physiologically relevant concentrations, to tryptophan metabolite activation than mouse AHR. Additionally, correlation analyses indicate a relationship linking major tryptophan metabolite abundance with AHR activity, suggesting these cecal/fecal metabolites represent biomarkers of intestinal AHR activity.

## Introduction

The defining feature of epithelial tissues (skin, reproductive, respiratory and gastrointestinal tracts) is to provide a barrier to the dynamic external environment, thus facilitating internal homeostasis. Despite this, ubiquitous colonization of apical barrier tissues by archaea, bacteria, fungi and viruses (collectively, the microbiota) is increasingly recognized as contributing vital aspects of normal host physiology.^1^ Such effects of the microbiota on host physiology are largely mediated through microbiota-derived factors rather than the microbiota *per se*. Host immune education (T- and B-cell repertoires) and surveillance occurs through the detection of microbial antigens or microbial-associated recognition patterns.^2^ Microbial metabolism of dietary constituents provides nutrients, e.g. vitamins, short-chain fatty acids and substrates, for host metabolism.^3^ Additionally, microbial detoxification of potential toxicants may confer protection to the host.^4^ Commensal microbiota also provide protection against pathogenic microbiota through nutrient competition and other mechanisms.^5,6^ It is likely that the microbiota community colonizing epithelial surfaces is somewhat self-regulating, although maintaining host-microbiota homeostasis also requires regulation by the host. Actually, the competing demands of a selectively permeable epithelial barrier require extensive, continuous sensing and regulation. Coincident with the appreciation of the importance of the microbiota has been the recognition of the aryl hydrocarbon receptor (AHR) as a pivotal regulator of epithelial barrier function, particularly within the gastrointestinal tract, where microbial burden and selective permeability are most evident. Previous studies have implicated the AHR as a sensor of microbially derived metabolites, particularly those derived from tryptophan catabolism and that such activation confers protection from chemical and microbial gastrointestinal challenge.^7,8^

The AHR is the only member of a family of environmental sensing transcription ligand-dependent transcription factors activated by an array of environmental contaminants, such as 2, 3, 7, 8-tetrachlorodibenzo-*p*-dioxin (TCDD) and polycyclic aromatic hydrocarbons (e.g. benzo(*a*)pyrene). Upon ligand binding, cytoplasmic AHR undergoes transformation and nuclear translocation which facilitates heterodimerization with the AHR nuclear translocator (ARNT/HIF1β) to form a competent transcription factor capable of inducing AHR target gene expression.^9^ AHR expression and activation within the gastrointestinal tract are demonstrated to influence gene expression associated with gastrointestinal function.

AHR is regarded as a principal regulator of xenobiotic metabolism, promoting the expression of the prototypical AHR target genes (e.g. CYP1A1, CYP1B1), involved in phase I and II metabolic detoxifications. Such expression confers both local and systemic protection from xenobiotics.^10^ AHR activation has been shown to profoundly influence systemic and tissue-specific immunity and is broadly considered immune-suppressive. However, closer examination reveals a more nuanced and complex action, dependent upon allelic variation, ligand, dose, species, tissue, etc. It is important to note that for mucosal immunity in the gastrointestinal tract, AHR activation increases the survival/residency/function of innate lymphoid group 3 cells (ILC3) and their RORγ-dependent production of IL22, a cytokine that regulates epithelial production of anti-microbial defensins, intestinal repair and maintenance of stem cell populations.^11–16^ Through both indirect, ILC3-dependent, and direct effects on the polarization of pro-inflammatory Th17 and anti-inflammatory Treg cell populations, AHR influences microbiota-dependent immune education/surveillance and inflammatory tone associated with gastrointestinal disease.^10,17^ In addition, the AHR promotes peripheral immune cell trafficking mediated by enhanced expression of chemokines and their cognate receptors (e.g. CCR6).^18,19^ Through indirect, direct, or combinatorial transcription factor recruitment/sequestration crosstalk mechanisms, AHR activation is demonstrated to influence the expression of diverse cytokines (e.g. IL1B, IL4, IL6, IL17, IL10, IL23, IL27, IFNγ, etc.).^19–23^ Such modulation of the cytokine milieu within the lamina propria of the intestine dictates the pro- or suppressive phenotype with consequences affecting barrier integrity and the community structure of the microbiota. In addition to promoting epithelial xenobiotic metabolism, AHR activity within the crypt-villi axis of the gastrointestinal tract regulates epithelial cell differentiation and repair.^11,24-26^ In combination, these and other AHR-mediated activities contribute to epithelial integrity and shape the immune/microbiota landscape of the gastrointestinal tract. Indeed, AHR knockout or toxicological models highlight the profound consequences of AHR expression and activation in the gastrointestinal tract.^27,28^ Environmental xenobiotic activation,^29^ dietary constituents containing pro-AHR ligands,^30,31^ or experimental high-dose AHR ligand models have been shown to activate the intestinal AHR in vivo. However, the primary source(s) of AHR activation within the gastrointestinal tract, especially under non-pathological conditions, is largely unknown.

Here we quantify a panel of tryptophan metabolites extracted from mouse cecal contents and human fecal matter, the latter from participants in a controlled clinical nutrition study. Additionally, we examine the role of the microbiota in the production of tryptophan metabolites and investigate the capacity of these metabolites to activate AHR signaling at physiologically relevant concentrations to assess which metabolites represent major sources of AHR activation in the gastrointestinal tract. Importantly, we also examine whether species-dependent differences exist between mouse/human AHR with regard to activation by microbiota-derived tryptophan metabolites, thus assessing whether mouse AHR activation corresponds to that of human AHR in the gastrointestinal tract.

## Materials and methods

### Chemicals and reagents

Indole-3-acetic acid and serotonin HCl were purchased from Alta Aesar (Ward Hill, MA, USA). All other tryptophan metabolites, HPLC grade chloroform and indole-d6 were purchased from Sigma–Aldrich (St. Louis, MO, USA) or Toronto Research Chemicals (North York, ON, Canada). All tryptophan metabolites were of the highest grade available (>96%). Indole was re-purified and verified by NMR as previously described.^32^ Analytical grade sodium hydroxide and sodium bicarbonate, as well as LC/MS grade solvents including methanol and acetonitrile, were purchased from Fisher Scientific (Hampton, NH, USA). The stock solutions of all reference standards for liquid chromatography were prepared in 10% acetonitrile (v/v) containing 1 µM chloropropamide (internal standard). The mixed standard solutions were gradually diluted with 10% acetonitrile (v/v) containing 1 µM chloropropamide for generating the calibration curves. The stock solutions of reference standards for GC were prepared in chloroform containing 30 µM indole-d6 (internal standard).

### Human stool

Two groups of stool samples were utilized in this study; the first set of 44 stool samples were from a controlled clinical nutrition diet study on a defined run-in diet.^33^ The second group was composed of 29 randomly obtained stool samples from BioIVT LLC (Hicksville, NY). All samples obtained were stored at −80°C.

### Cecal contents collection from conventional and GF mice

C57BL/6 J wild type mice were originally purchased from Jackson Laboratories (Bar Harbor, MN). Germ-free (GF) C57BL/6 J mice were from the Pennsylvania State University Gnotobiotic Animal Research Facility. Mice were bred in-house and fed on a standard animal chow diet. Animal experiments were performed after approval by the Institutional Animal Care and Use Committee. Fresh cecal contents from conventional and GF mice were collected and stored at −80°C.

### Global profiling by UPLC–Orbitrap Fusion–MS

Cecal contents (~50 mg) from conventional and GF mice or 30 mg of freeze-dried human stool were mixed with 1 ml of ice-cold 80% methanol/0.1% formic acid (v/v). Each mixture was homogenized with 8–10 1 mm zirconium beads using a BeadBlaster^TM^ 24 (Benchmark Scientific, Edison, NJ) homogenizer. All samples were homogenized according to the program parameters: 6500–1 × 30–005 (three times). After vortexing, samples were sonicated for 20 min in an ice water bath. Samples centrifuged at 20,000 × g for 20 min at 4°C. The supernatants were collected, dried in a Savant SpeedVac (Thermo Scientific, Waltham, MA, USA) and reconstituted in 100 μl of 3% MeOH (v/v) containing 1 µM chlorpropamide (internal standard), vortexed and sonicated for about 20–25 min, the samples were centrifuged at 10,000 × g at 4°C for 15 min. Samples were then analyzed using a Vanquish UHPLC system connected to an Orbitrap Fusion Tribrid MS (Thermo Fisher Scientific, Waltham, MA, U.S.A.). Further details of chromatographic conditions have been previously described.^34^ Differential analyses were performed by the use of Compound Discoverer (Thermo Fisher Scientific, Waltham, MA, U.S.A.) or in the case of human fecal analysis, MS-DIAL (Ver. 3.08) and its database were used in the data analyses.^35,36^ The identification accuracies were calculated between standards and either cecal or fecal extracts using Stein and Scott’s composite similarity score.^37^ The untargeted global profiling datasets were deposited in UCSD Metabolomics Workbench, a resource sponsored by the Common Fund of the National Institutes of Health. A description of the project was also included. The data track ID for global profiling for human feces and cecal contents were labeled as 1957 (Global profiling for human feces (dongf01_20200326_184856) DATATRACK_ID) and 1958 (Global profiling for cecal contents (dongf01_20200326_193205) DATATRACK_ID) respectively.

### Targeted quantification of compounds by UPLC-QTOF-MS

Mouse cecal contents or human stool were weighed and mixed with extraction solvent of ice-cold 50% acetonitrile (v/v) containing 1 µM chloropropamide in 1/2 ratio (Ws/Vs). The mixture was homogenized with 1 mm zirconium beads using a BeadBlaster^TM^ 24 homogenizer followed by sonication for 20 min in an ice bath. All samples were homogenized according to the program parameters: 6500–1 × 30–005 (×3). Following centrifugation at 20,000 × g at 4°C for 20 min, the supernatants were collected and diluted by 5-fold and 50-fold with pure water containing 1 µM chlorpropamide before being subjected to analysis. Targeted quantitation was performed to determine the concentration of tryptophan metabolites from mouse cecal contents or human stool by reverse-phase UHPLC using a Prominence 20 UFLCXR system (Shimadzu, Columbia, MD, USA) with a Waters (Milford, MA, USA) BEH C18 column (2.1 × 100 mm × 1.7 µm particle size) maintained at 55°C and a 20 min aqueous acetonitrile gradient, at a flow rate of 250 µL/min. Solvent A was HPLC grade water with 0.1% formic acid and solvent B was HPLC grade acetonitrile with 0.1% formic acid. The initial conditions were 97% A and 3% B, increasing to 45% B at 10 min, 75% B at 12 min where it was held at 75% B until 17.5 min before returning to the initial conditions. The eluate was delivered into a 5600 (QTOF) TripleTOF using a Duospray™ ion source (AB Sciex, Framingham, MA, USA). The capillary voltage was set at 5.5 kV in positive ion mode with a declustering potential of 80 V. The mass spectrometer was operated with a 100 ms TOF scan from 50 to 500 m/z, and 16 MS/MS product ion scans (100 ms) per duty cycle using a collision energy of 50 V with a 20 V spread.

### Indole and 3-methylindole quantification by GC-MS

Sample processing protocols for cecal contents were according to the method described with some minor modifications.^38^ Briefly, ~30 mg of fresh cecal content was mixed with 300 μL of 1 M NaOH solution, homogenized with 1 mm zirconium beads and centrifuged at 16,000 × g at 4°C for 20 min. A volume of 200 μL of supernatant was collected into an autosampler vial. Then, the residue was re-extracted with 200 μL of cold methanol with the same steps of homogenization and centrifugation. The first supernatant in the sample vial was combined with 200 μL of supernatant from the second extraction. Subsequently, 300 μL of chloroform containing 30 µM indole-d6 was added to the mixed supernatant and samples were shaken for 10 s. Another 400 μL of 50 mM sodium bicarbonate solution was added again, and samples were shaken for another 10 s. A volume of 100 μL chloroform phase was transferred to GC vials for further analysis after centrifugation at 2000 × g for 10 min at 4°C. Indole and 3-methylindole were quantified with an Agilent 7890A gas chromatograph coupled with an Agilent 5975 mass spectrometer (Agilent Technologies Santa Clara, CA, USA).

### Quantification limit and linearity

The limit of quantification (LOQ) represents the lowest concentration of analyte which can be quantitatively determined and obtained by analyzing the signal-to-noise (S/N) ratio of 10 performed by PeakView (AB Sciex, Framingham, MA, USA) software for LC/MS, Enhanced ChemStation (Agilent MSD ChemStation) for GC/MS. The regression parameters of slope, intercept and correlation coefficient were calculated by linear regression modeling using seven serial dilutions of standard mix solutions containing internal standard of chlorpropamide at 1 µM (Table S3-5).

### Recovery and matrix effect

We wanted to account for differences in water content and matrix composition in each sample. This was accomplished by measuring a known amount of analytes spiked into the matrix to assess recovery. In this study, pooled cecal contents or human stool were chosen and divided into three sections. Two of those were, respectively, spiked with an amount of standard mix solution equal to calculated quantity of AHR-related compounds in cecal contents or human stool before and after extraction process. The third section was used to determine endogenous concentrations of compounds in pooled cecal contents or human stool. Then recovery could be investigated as follows: Recovery = (Peak area of the spiked analyte before extraction – Peak area of the endogenous analyte)/(Peak area of the spiked analyte after extraction – Peak area of the endogenous analyte). The matrix effect is caused by the components coextracted with the analytes which could suppress or enhance the signal response of target analytes.^39,40^ Matrix effect could lead to poor analytical accuracy of quantification of an analyte. Here, matrix effect was evaluated by comparing the response of mixed standards solution in pure water with that of mixed standards solution in pooled cecal contents or human stool. The matrix effect was achieved according to the following formula: Matrix effect = (Peak area of the spiked analyte in cecal contents or human stool – Peak area of the endogenous analyte in cecal contents)/Peak area of the spiked analyte in water.

### Cell-based reporter assay-Tryptophan metabolites

HepG2 40/6 was generated as previously described,^41^ and Hepa 1.1 cells were a gift from Dr. Michael S. Denison. Cells were seeded in 12-well plates and cultured as described.^42^ Cells were treated with TCDD or tryptophan metabolites in DMSO, 0.1% final concentration in cell culture. After 4 h incubation, cells were lysed with 200 μL of lysis buffer consisting of 25 mM Tris-phosphate (pH 7.8), 2 mM dithiothreitol, 2 mM 1,2-diaminocyclohexane-N,N,N’,N’-tetraacetic acid, 10% (v/v) glycerol and 1% (v/v) Triton X-100, and stored at −80°C until analysis. As per the manufacturer’s instructions, luciferase activity was determined using a TD-20e luminometer and luciferase assay substrate (Promega, Madison, WI, USA).

### Cell-based reporter assay-fecal extracts

HepG2 40/6 cells were treated with 200-fold diluted fecal extracts from the supernatants for targeted quantitation, and luciferase level was measured after 4 h with the same protocol used for tryptophan metabolites.

### RNA isolation and RT-qPCR

RNA was isolated from Hepa1 and Caco2 cells treated with tryptophan metabolites at the concentration indicated and quantitative real-time PCR (RT-qPCR) was performed as previously described.^30^

### Statistical analysis

Concentrations of tryptophan metabolites were normalized to cecal or fecal wet weight. All data were compared using either Student’s *t* test, one-way or two-way analysis of variance with Tukey multiple comparison posttest, where appropriate, in GraphPad Prism 6.01 (GraphPad Software, Inc, La Jolla, CA, USA) to determine statistical significance between different groups. The value of *p* < .05 was considered statistically significant (*p < .05; **p < .01; ***p < .001; ****p < .0001).

## Results

### Discovery, validation of tryptophan metabolites in mouse cecal contents/human stool and assessment as AHR activators

Although the essential amino acid tryptophan itself is neither an AHR ligand nor activator, it is recognized as a precursor for host and microbiota-dependent synthesis of AHR ligands. The luminal contents of the gastrointestinal tract provide a rich source of tryptophan and microbial metabolic activity and thus represent a reservoir of potential AHR activity. Methanolic extracts of freeze-dried cecal contents from both conventional and GF C57BL6/J mice fed a standard chow diet were analyzed using an untargeted mass spectrometry-based metabolomic approach, in an attempt to characterize the microbiota-dependent tryptophan metabolite profile within the cecum with regard to potential AHR activity. Utilizing such an untargeted approach combined with Compound Discoverer^TM^ deconvolution analysis provided the opportunity to potentially identify unreported AHR-sensitive tryptophan metabolites in conjunction with those expected to be present and dependent on the microbiota. Analysis between conventional and GF cecal extracts reported more than 500 significantly different features, highlighting the input of the microbiota in determining the cecal metabolome. The indole moiety was selected as a screening criterion to putatively identify the subset of tryptophan-dependent metabolites within the set of significant *m/z* features. Using this approach, two candidate features were selected for LC/MS/MS validation with commercially available standards. Retention time, MS/MS spectra and similarity scores (>600) confirmed the identity of 3-indoleacrylic acid and indole-3-carboxylic acid, as specifically detectable in cecal contents from conventionally housed mice when compared to GF counterparts (Figures S1-S2).

A similar approach was applied to the analysis of potentially novel tryptophan metabolites in human fecal matter. Methanolic extracts derived from freeze-dried human stool obtained from a defined feeding study were subjected to mass spectrometry-based metabolomics and subsequent MS-DIAL deconvolution analysis, which reported ~500 features; of these, 52 features were either annotated or identified.^33^ Two novel putative tryptophan-derived AHR ligands, 3-methyl-2-oxindole and 5-hydroxyindole-3-acetic acid, were selected for LC/MS validation with commercially available standards. Retention time, MS/MS spectra and similarity scores (>600) confirmed the identity of 3-methyl-2-oxindole and 5-hydroxyindole-3-acetic acid, as detectable in human fecal matter (Figures S1-S2).

**Figure 1.**
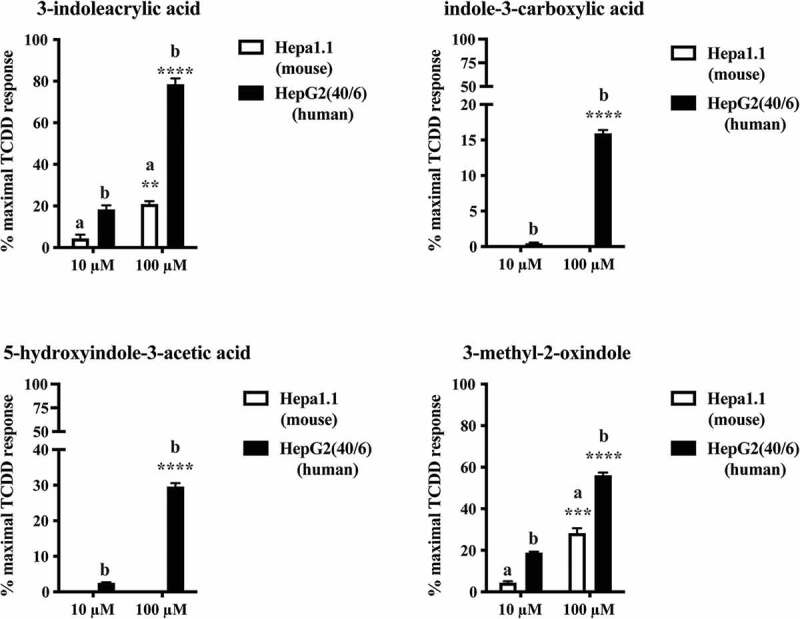
Identification of tryptophan metabolites as novel AHR activators. HepG2 40/6 or Hepa 1.1 cells were treated with each novel tryptophan metabolites identified in either cecal or fecal material to assess AHR activation potential. Cells were lyzed after 4 h and luciferase activity determined. Due to cell line dependent differential basal activity and AHR-dependent induction, data represent mean luciferase activity (background subtracted) expressed as percentage of maximal TCDD-dependent activity. The data are mean ± SEM, ** *P* < .01, *** *P* < .001, **** *P* < .0001.

To assess their potential to activate AHR, each of these four candidate tryptophan metabolites identified in mouse cecal content or human stool was examined in the context of both mouse (Hepa 1.1) and human (HepG2 40/6) AHR-dependent reporter assay systems (Figure 1). When expressed relative to the maximal response achieved with the prototypical AHR ligand TCDD (10 nM), each metabolite demonstrated a capacity to stimulate human AHR activity in a dose-dependent manner (10 and 100 μM) with a potency rank order of 3-indoleacrylic acid >3-methyl-2-oxindole >5-hydroxyindole-3-acetic acid > indole-3-carboxylic acid. Relative to human AHR, the capacity of 3-indoleacrylic acid and 3-methyl-2-oxindole to stimulate mouse AHR activity is markedly suppressed while indole-3-carboxylic acid and 5-hydroxyindole-3-acetic acid fail to elicit AHR activity.

With the exception of 3-methyl-2-oxindole, each of the four-candidate tryptophan-derived putative AHR ligands has been characterized as being detectable in human stool in the Human Metabolome Database (HMDB).^43^ These data suggest that these tryptophan metabolites represent novel, unreported AHR activators detectable in cecal and fecal matter and thus may contribute to AHR activity within the gastrointestinal tract, especially with regard to human AHR.

### Quantification of tryptophan metabolites in mouse cecal contents

**Figure 2.**
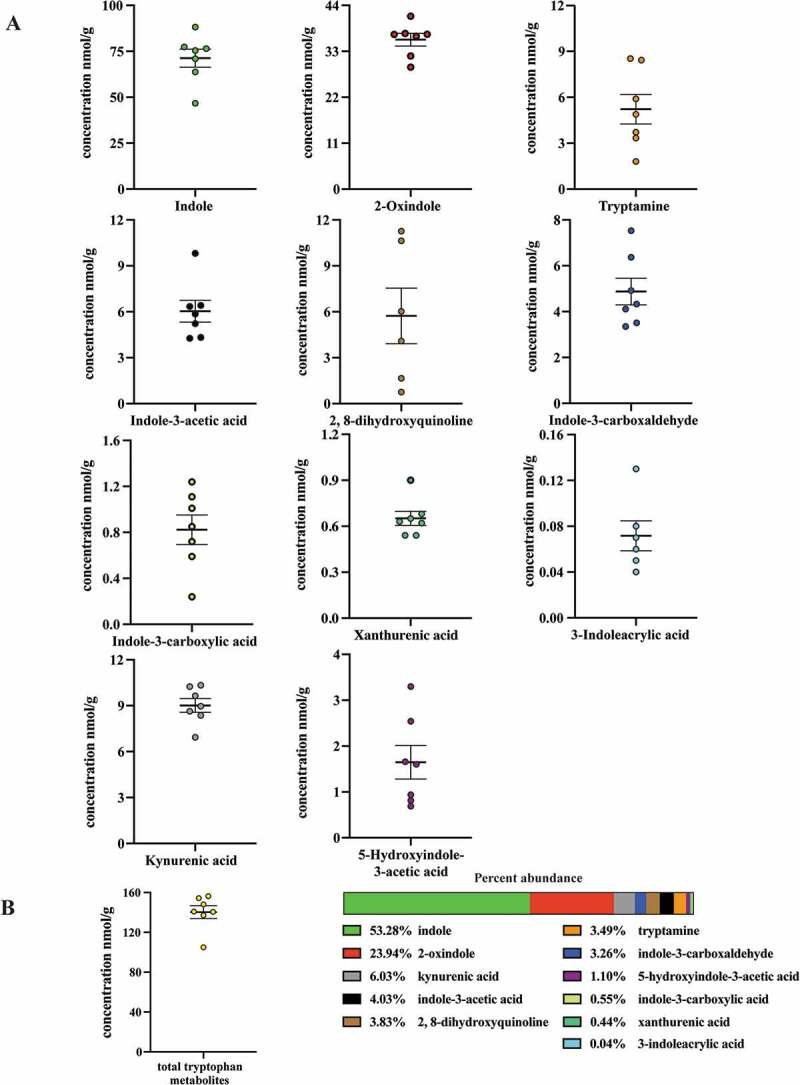
Level of 11 tryptophan metabolites in mouse cecal contents. LC-MS and GC-MS analysis was utilized to quantitate tryptophan metabolites in cecal extracts. (a) Concentration of seven individual tryptophan metabolites in cecal contents of mice on a chow diet. (b) Overall concentration of all measured tryptophan metabolites and percent abundance of each metabolite relative to the overall tryptophan metabolite levels. The data are mean ± SEM.

Having detected (ex vivo from mouse cecal contents and human stool) and identified 3-methyl-2-oxindole and 5-hydroxyindole-3-acetic acid, 3-indoleacrylic acid and indole-3-carboxylic acid as novel in vitro AHR activators, we wished to examine their respective AHR activities within a more physiologically relevant context. To achieve this we quantified these four metabolites in conjunction with a panel of additional tryptophan metabolites (indole-3-acetic acid, tryptamine, serotonin, 2, 8-dihydroxyquinoline, kynurenic acid xanthurenic acid, 2-oxindole, indole-3-carboxaldehyde, 3-methylindole and indole), all of which have been previously detected within the gastrointestinal tracts of mice and humans and reported to directly activate AHR signaling. However, serotonin appears to be an indirect ligand through inhibition of CYP1A1 activity.^44^ All compounds were quantified by LC/MS with the exception of indole, which by virtue of its high volatility was quantified by GC/MS. Sample extracts derived from fresh mouse cecal contents were quantified through interpolation of standard curves and corrected for recovery and matrix effects. Cecal contents were chosen for analysis due to being the major initial site of bacterial metabolism. Analyses of mouse cecal contents (Table 1, Figure 2) revealed indole to be the most abundant tryptophan metabolite, with a concentration of ~80 nmol/g, representing >50% by mass of the metabolites examined. 2-oxindole, with a concentration of ~36 nmol/g, represented ~24% of the tryptophan metabolite mass. The remaining tryptophan metabolites, representing ~25% of the tryptophan metabolite mass exhibited concentrations ranging from below the limit of detection (3-methylindole), to 0.06, 0.65, 0.82, 1.65, 4.88, 5.22, 5.73, 6.03 and 9.01 nmol/g; 3-indoleacrylic acid, xanthurenic acid, indole-3-carboxylic acid, 5-hydroxyindole-3-acetic acid, indole-3-carboxaldehyde, tryptamine, 2, 8-dihydroxyquinoline, indole-3-acetic acid and kynurenic acid, respectively. These data indicate that the novel AHR activators, 3-indoleacrylic acid and indole-3-carboxylic acid, while detectable in mouse cecal contents, represent <0.5% by mass of the examined metabolites. Similarly, those novel AHR activators, 5-hydroxyindole-3-acetic acid, 3-methyl-2-oxindole, originally detected in human stool, represent ~1% by mass in the case of 5-hydroxyindole-3-acetic acid or below the limit of quantification in the case of 3-methyl-2-oxindole. Such data suggest that the newly identified novel AHR activators, 5-hydroxyindole-3-acetic acid, indole-3-carboxylic acid and 3-indoleacrylic acid, are minor tryptophan metabolites and thus unlikely to manifest as a significant source of AHR activation in the mouse gastrointestinal tract.

**Table 1.** Quantification of Trp metabolites in cecal contents of pathogen-free mice.**^a.^**

Compound	Concentration^b^ (nmol/g)
Indole-3-acetic acid	6.03 ± 0.66
3-Indoleacrylic acid	0.06 ± 0.01
Tryptamine	5.22 ± 0.89
Serotonin	0.98 ± 0.15
Indole-3-carboxaldehyde	4.88 ± 0.54
2,8-Dihydroxyquinoline	5.73 ± 1.66
Kynurenic acid	9.01 ± 0.42
Xanthurenic acid	0.65 ± 0.04
2-Oxindole	35.79 ± 1.40
Indole-3-carboxylic acid	0.82 ± 0.12
5-Hydroxyindole-3-acetic acid	1.65 ± 0.34
Indole	79.66 ± 5.11

**^a^**: Both 3-methyl indole and 3-methyl-2-oxindole were below LOQ. **^b^**: Corrected concentration of each compound equals observed concentration divided by recovery and matrix effect.

### Generation of cecal tryptophan metabolites is dependent upon the microbiota

Having identified, quantified and validated the AHR activation potential of tryptophan metabolites detectable in mouse cecal contents or human stool, we next investigated whether these metabolites are derived from the diet, host, microbial activity or some combination of the three. Comparison between quantifiable cecal tryptophan metabolites from conventionally housed C57BL6/J mice and GF counterparts, maintained on identical diets, revealed markedly different metabolite profiles (Table 2 and S3). With the exception of indole-3-carboxylic acid and 5-hydroxyindole-3-acetic acid, the level of all tryptophan metabolites was significantly (*p* < .05) reduced in GF mice. Many of the metabolites, including indole, 2-oxindole and indole-3-acetic acid, were below the limit of quantification. These data suggest that, in mice, a viable microbiota is a necessary requirement for the synthesis of AHR-sensitive cecal tryptophan metabolites.

**Table 2.** Comparison of Trp metabolite concentrations in pathogen-free and GF mice.

Compound	Conventional mice Mean (nmol/g)	GF mice Mean (nmol/g)
Indole-3-acetic acid	6.03 ± 0.66	0.16 ± 0.07^a^
3-Indoleacrylic acid	0.06 ± 0.01	< LOQ
Tryptamine	5.22 ± 0.89	< LOQ
Serotonin	0.98 ± 0.15	< LOQ
Indole-3-carboxaldehyde	4.88 ± 0.54	0.43 ± 0.01^a^
2,8-Dihydroxyquinoline	5.73 ± 1.66	< LOQ
Kynurenic acid	9.01 ± 0.42	0.50 ± 0.02^a^
Xanthurenic acid	0.65 ± 0.04	< LOQ
2-Oxindole	35.79 ± 1.40	< LOQ
Indole-3-carboxylic acid	0.82 ± 0.12	0.85 ± 0.17
5-Hydroxyindole-3-acetic acid	1.65 ± 0.34	0.78 ± 0.17
Indole	79.66 ± 5.11	< LOQ

^a^: Significant difference (*p* < 0.05) between GF and pathogen-free mice.

### Quantification of tryptophan metabolites in human stool

**Figure 3.**
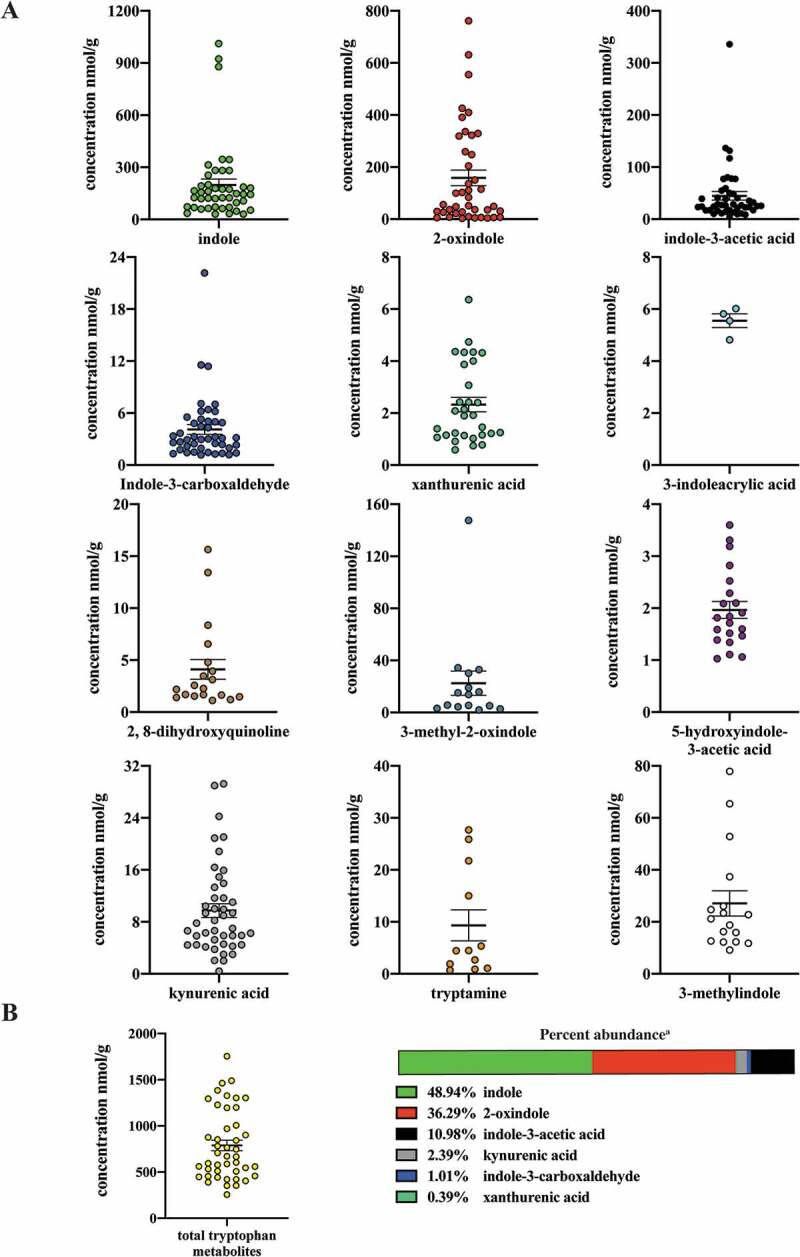
Level of 12 tryptophan metabolites in human feces. LC-MS and GC-MS analysis was utilized to quantitate tryptophan metabolites in fecal extracts derived from humans on a defined diet. The data are mean ± SEM.

Next we examined tryptophan metabolite profiles in human stool. To mitigate diet-dependent modulation of the panel of tryptophan metabolites, similar quantitative analyses were performed upon human fecal matter obtained from a defined feeding study, i.e. all participants consumed the same diet. Similar to the quantitative profile obtained with mouse cecal contents, indole and 2-oxindole exhibited the highest mean abundance in human stool of 199 and 158 nmol/g, respectively, and together represent >80% by mass within the panel of selected tryptophan metabolites (Table 3, Figure 3). Both indole and 2-oxindole were quantifiable in >90% of fecal samples but exhibited variation (30.14–1011.12 and 0–761.88 nmol/g, respectively) across samples. The median abundance of indole and 2-oxindole was 145 and 50 nmol/g, respectively. Notable differences between mouse cecal contents and human stool were observed in the quantification and rank order of tryptophan metabolite abundance. However, a direct comparison between the amount of specific tryptophan metabolites between species may be difficult to interpret. 3-methyl indole and 3-methyl-2-oxindole were quantifiable in human stool but not in mouse cecal content. In contrast, indole-3-carboxylic acid was quantifiable in mouse cecal content but not in human fecal matter. A number of the tryptophan metabolites exhibited selective representation across the pool of human fecal samples, 3-methylindole and 2, 8-dihydroxyquinoline were represented in 38% and 43% of samples, respectively. With regard to the newly identified novel AHR activators, indole-3-acrylic acid, 3-methyl-2-oxindole and 5-hydroxyindole-3-acetic acid, each was quantifiable in only 9%, 34% and 47% of human fecal samples, respectively. The novel AHR activator, indole-3-carboxylic acid, originally identified from mouse cecal contents, was below the limit of quantification (0.17 nmol/g) in all human fecal samples. Consequently, for a number of tryptophan metabolites, a comparison between mean and median abundance yields marked differences.

**Table 3.** Quantification of Trp metabolites in defined diet human feces.**^a.^**

Compound	*n*	Mean concentration^b^ (nmol/g)	Range (nmol/g)	Median concentration (nmol/g)
indole-3-acetic acid	44	44.64 ± 54.87	4.30–335.74	26.32
3-indoleacrylic acid	4	5.55 ± 0.53	<LOQ-6.01	<LOQ
tryptamine	12	9.32 ± 10.33	<LOQ-27.68	<LOQ
serotonin	4	22.12 ± 3 2.31	<LOQ-70.57	<LOQ
indole-3-carboxaldehyde	44	4.11 ± 3.69	1.16–22.17	3.05
2,8-dihydroxy- quinoline	19	4.11 ± 4.16	<LOQ-15.63	<LOQ
kynurenic acid	44	9.74 ± 6.96	0.42–29.24	7.39
xanthurenic acid	30	2.33 ± 1.52	<LOQ-6.36	1,15
2-oxindole	41	158.36 ± 191.68	<LOQ-761.88	50.18
3-methyl-2-oxindole	15	22.47 ± 36.39	<LOQ-147.58	<LOQ
5-hydroindole-3-acetic acid	21	1.97 ± 0.75	<LOQ-3.60	<LOQ
indole	44	199.0 ± 219.46	30.14–1011.12	144.66
3-methylindole	17	27.09 ± 20.02	<LOQ-77.92	<LOQ

**^a^**: Indole-3-carboxylic acid < LOQ.**^b^**: Concentration of each compound equals observed concentration divided by recovery and matrix effect and represents mean ± SEM concentration across only those samples quantified as >LOQ.

**Figure 4.**
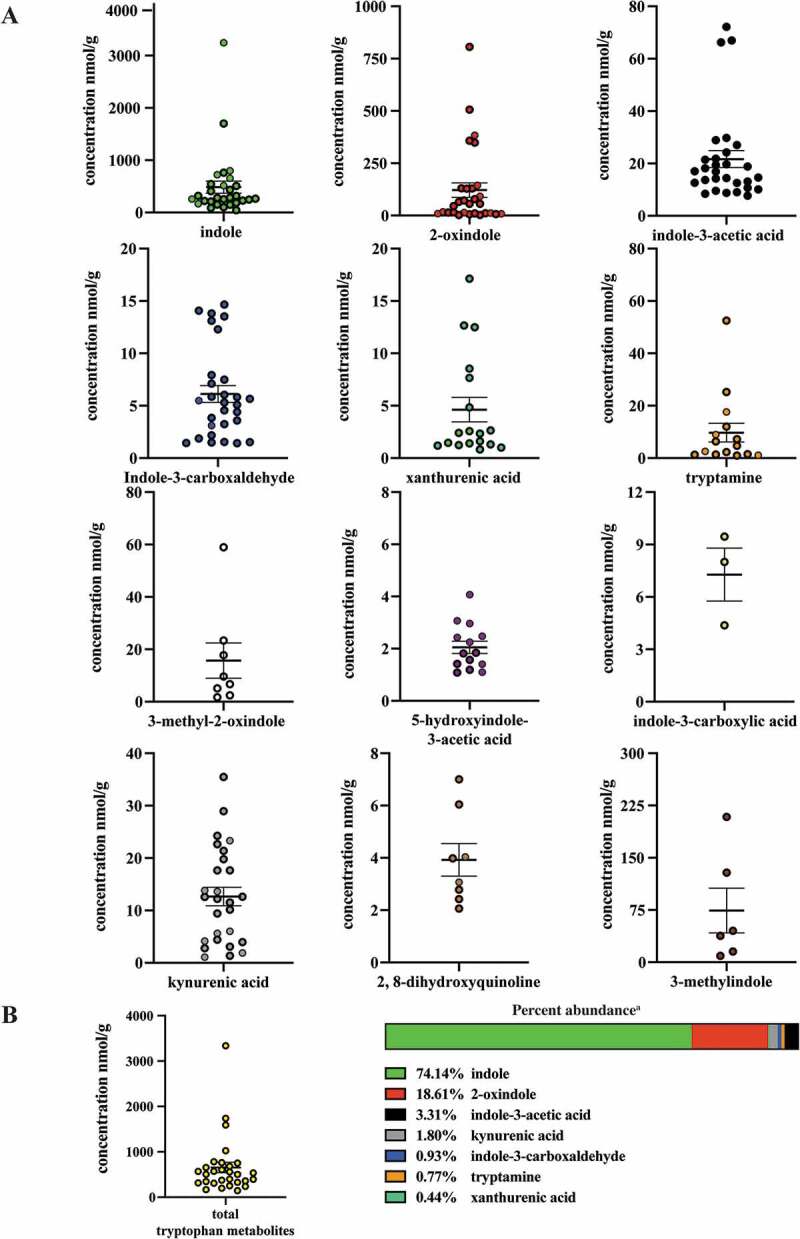
Level of 12 tryptophan metabolites in human feces. LC-MS and GC-MS analysis was utilized to quantitate tryptophan metabolites in fecal extracts derived from humans on an ad libitum diet. The data are mean ± SEM.

The quantification of AHR-sensitive tryptophan metabolites in human fecal samples obtained from subjects on a defined feeding study allowed the establishment of quasi-baseline concentrations without confounding dietary factors and permitted a comparison with human fecal samples obtained from subjects with unrestricted, ad libitum dietary consumption (Table 4, Figure 4). Analyses of ad libitum fecal samples identified significant differences in the mean abundance of some tryptophan metabolites which were quantifiable in >50% of all samples. Fecal indole and xanthurenic acid levels were significantly (*p* < .01 and *p* < .05, respectively) higher in the ad libitum samples compared to those obtained from the defined feeding study. Conversely, the level of fecal indole-3-acetic acid was significantly (*p* < .05) lower in the ad libitum samples compared to the defined population. The remaining tryptophan metabolites, quantifiable in >50% of all samples, did not exhibit significant differences in abundance. However, for some metabolites, the frequency of representation across defined and ad libitum samples was different. The novel AHR activator 3-indoleacrylic acid was quantifiable in 9% of defined samples but not quantifiably represented in any ad libitum samples. Conversely, indole-3-carboxylic acid was quantifiable in 10% of ad libitum samples but not quantifiably represented in any defined samples. The frequency of representation for 3-methyl-2-oxindole and 5-hydroxyindole-3-acetic acid was similar in both fecal cohorts. The data presented in Tables 1, 3 and 4 have been compiled into a single table in order to allow direct comparisons of individual tryptophan metabolites to be made (Table 5).

**Table 4.** Quantification of Trp metabolites in ad lithium diet human feces**^a.^**

Compound	*n*	Mean concentration^b^ (nmol/g)	Range (nmol/g)	Median concentration (nmol/g)
indole-3-acetic acid	29	21.66 ± 17.29	7.67–72.15	16.89
tryptamine	15	9.71 ± 13.76	<LOQ-52.53	0.95
serotonin	7	11.52 ± 6.22	<LOQ-18.60	<LOQ
indole-3-carboxaldehyde	29	6.12 ± 4.31	1.43–14.66	5.30
2,8-dihydroxy- quinoline	8	3.93 ± 1.76	<LOQ-7.0	<LOQ
kynurenic acid	27	12.65 ± 9.21	<LOQ-35.48	11.52
xanthurenic acid	18	4.63 ± 4.95	<LOQ-17.13	1.25
2-oxindole	29	121.90 ± 186.88	2.58–806.07	60.00
indole-3-carboxylic acid	3	7.28 ± 2.62	<LOQ-9.46	<LOQ
3-methyl-2-oxindole	8	15.72 ± 19.04	<LOQ-58.98	<LOQ
5-hydroxyindole-3-acetic acid	14	2.05 ± 0.88	<LOQ-4.07	<LOQ
indole	29	485.53 ± 622.18	45.37–3245.0	280.16
3-methlyindole	6	74.17 ± 7 8.62	<LOQ-208.68	<LOQ

**^a^**:3-indoleacrylic acid was < LOQ. **^b^**: Concentration of each compound equals observed concentration divided by recovery and matrix effect and represents mean ± SEM concentration across only those samples quantified as >LOQ.

**Table 5.** A compilation of the data presented in Table 1, 3 and 4.

Tryptophan metabolite	Mean abundance nmole/g^a^					
	Mouse cecal (conventional)	Mouse cecal (germ-free)	Mouse cecal (purified diet)	Mouse cecal (purified to chow diet)	Human feces (defined diet cohort)	Human feces (ad libitum diet cohort)
Indole	79.7	<LOQ	40.4	70.1	198.9	485.5
2-oxindole	35.8	<LOQ	15.6	23.4	147.6	121.9
Kynurenic acid	9.0	0.5	3.1	4.9	9.7	11.8
Indole-3-acetic acid	6.0	0.2	4.2	3.2	44.6	21.7
Dihydroxyquinoline	5.7	<LOQ	nd^b^	nd^b^	<LOQ	<LOQ
Tryptamine	5.2	<LOQ	nd^b^	nd^b^		5.0
Indole-3-carboxaldehyde	4.9	0.4	nd^b^	nd^b^	4.1	6.1
5-hydroxyindole-3-acetic acid	1.6	0.8	nd^b^	nd^b^	<LOQ	<LOQ
Indole-3-carboxylic acid	0.8	0.9	nd^b^	nd^b^	<LOQ	<LOQ
Xanthurenic acid	0.7	<LOQ	nd^b^	nd^b^	1.6	2.87
3-indoleacrylic acid	0.1	<LOQ	nd^b^	nd^b^	<LOQ	<LOQ
3-methylindole	<LOQ	<LOQ	nd^b^	nd^b^	<LOQ	<LOQ
3-methyl-2-oxindole	<LOQ	<LOQ	nd^b^	nd^b^	<LOQ	<LOQ

^a^> LOQ representation in >50% of samples

^b^not determined

### Efficacy of cecal/fecal tryptophan metabolites as mouse and human AHR activators

**Figure 5.**
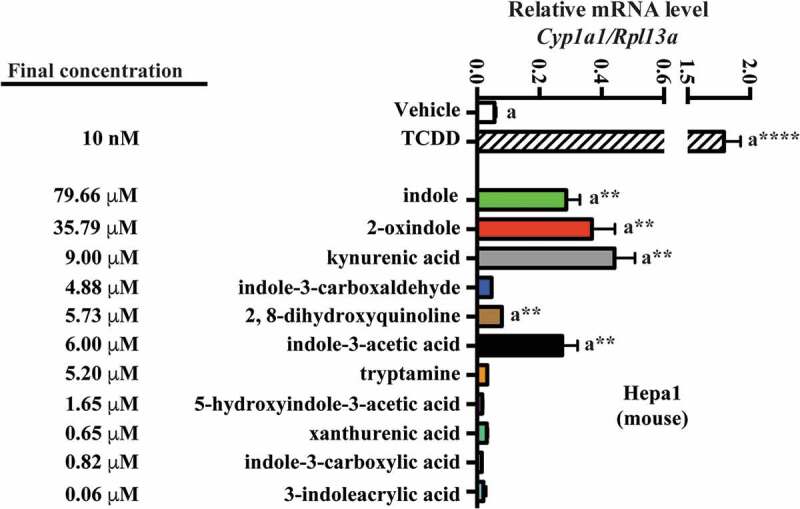
Assessment of AHR transcriptional activity mediated by tryptophan metabolites. Hepa 1 cells were treated for 4 h with tryptophan metabolites at the concentration observed in cecal contents. The data are mean ± SEM, ** *P* < .01, **** *P* < .0001.

**Figure 6.**
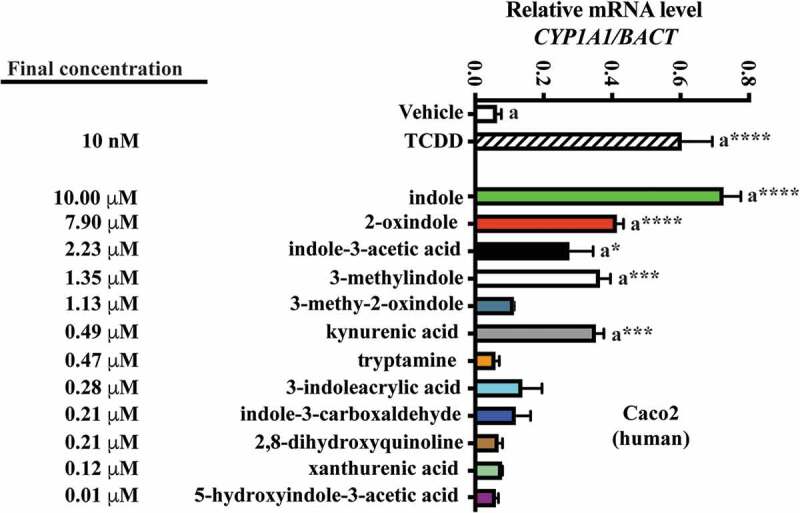
Assessment of AHR transcriptional activity mediated by tryptophan metabolites. Caco2 cells were treated for 4 h with tryptophan metabolites at the concentration observed in defined diet human feces. The data are mean ± SEM, *** *P* < .001, **** *P* < .0001.

To address the relative efficacy, with regard to mouse and human AHR activation, of each of the novel tryptophan-derived AHR activators detected in cecal/fecal matter, in conjunction with those previously reported as activating AHR, we adopted a quantitative gene expression approach, using the prototypical AHR target gene *Cyp1a1* (mouse) or *CYP1A1* (human) as outputs for AHR activity (Figures 5 and 6).

Mouse Hepa 1 cells were exposed to vehicle for 4 h, then 10 nM TCDD, or each of the indicated tryptophan metabolites, and *Cyp1a1* mRNA was quantified (Figure 5). The concentration of each metabolite examined reflects those obtained through LC/MS and GC/MS quantification of mouse cecal contents. In Hepa1 cells, the indicated tryptophan metabolites exhibited non-saturable and variable levels of *Cyp1a1* inducibility. At the concentrations tested, indole, 2-oxindole, kynurenic acid and indole-3-acetic acid elicited significant *Cyp1a1* induction relative to vehicle (~5-fold, *p* < .01). 2, 8-dihydroxyquinoline induced *Cyp1a1* to a lesser yet significant extent (1.3-fold, *p* < .01). None of the remaining tryptophan metabolites elicited significant *Cyp1a1* inducibility.

A similar approach was adopted to examine human fecal tryptophan metabolites and human AHR activation in Caco2 cells (Figure 6). Consistent with a reduced affinity of human AHR for TCDD compared to mouse AHR, *CYP1A1* inducibility in response was relatively diminished. However, due to the enhanced activation potential of the human AHR for indole-derived compounds,^32^ we further diluted these concentrations by 20-fold to restrict saturable *CYP1A1.* And yet, in Caco2 cells, indole still elicited a significant saturation of *CYP1A1* expression (~15-fold, *p* < .001), while the remaining tryptophan metabolites elicited non-saturable yet variable levels of *CYP1A1* induction. 2-oxindole, indole-3-acetic acid, 3-methyl indole and kynurenic acid prompted significant *CYP1A1* inducibility. The remaining tryptophan metabolites, however, failed to elicit significant induction. Comparison between the Hepa1 and Caco2 induction profiles indicates commonality across mouse and human AHR with regard to activation by the panel of tryptophan metabolites at the concentrations tested. In order to have confidence in comparing the results in two different cell lines and species, a time course experiment in Caco2 and Hepa 1 cells was performed to establish whether the cell lines exhibit similar kinetics of induction and the time of maximum induction. Caco2 and Hepa 1 cells were treated with kynurenic acid at the concentrations utilized in Figures 5 and 6. The results reveal both similar kinetics of induction and a maximal level of induction at 4 h (Fig. S4).

### AHR activation potential in human stool and correlation with fecal tryptophan metabolites

**Figure 7.**
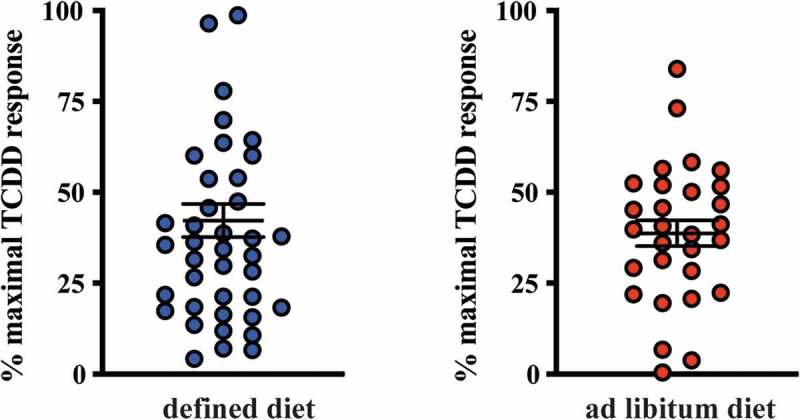
Relative AHR transcriptional activity induced by human fecal extracts. HepG2 40/6 cells were treated with fecal extracts from defined and Ad libitum diet groups and the level of AHR transcriptional activity assessed. The data are mean ± SEM.

**Figure 8.**
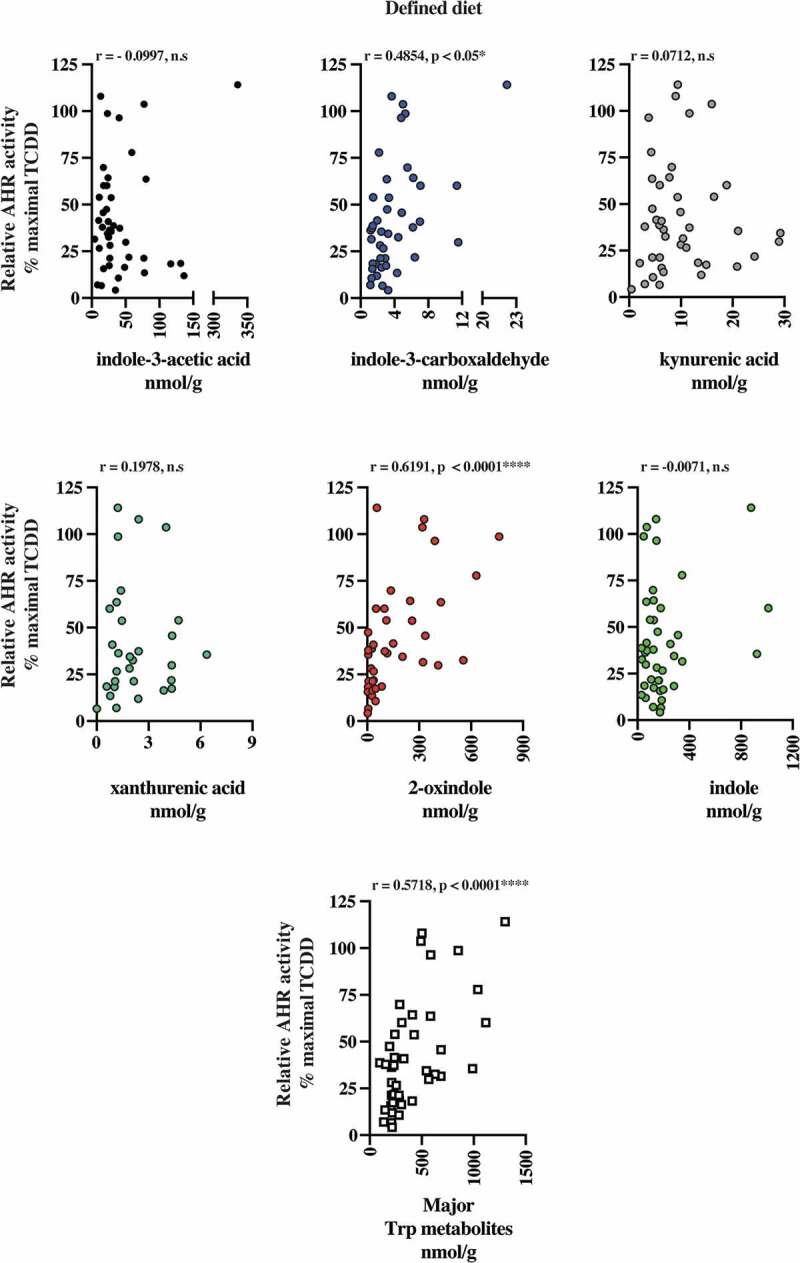
2-oxindole and major tryptophan metabolites levels pooled together correlated with induced AHR transcriptional activity in human fecal extracts from the defined diet group. Six tryptophan metabolites that were present in more than 50% of the fecal samples and were correlated individually with AHR transcriptional activity induced HepG2 40/6 cell by fecal extracts from each individual. Each graph contains the Pearson *r* value correlation coefficient and two-tailed *p* value.

**Figure 9.**
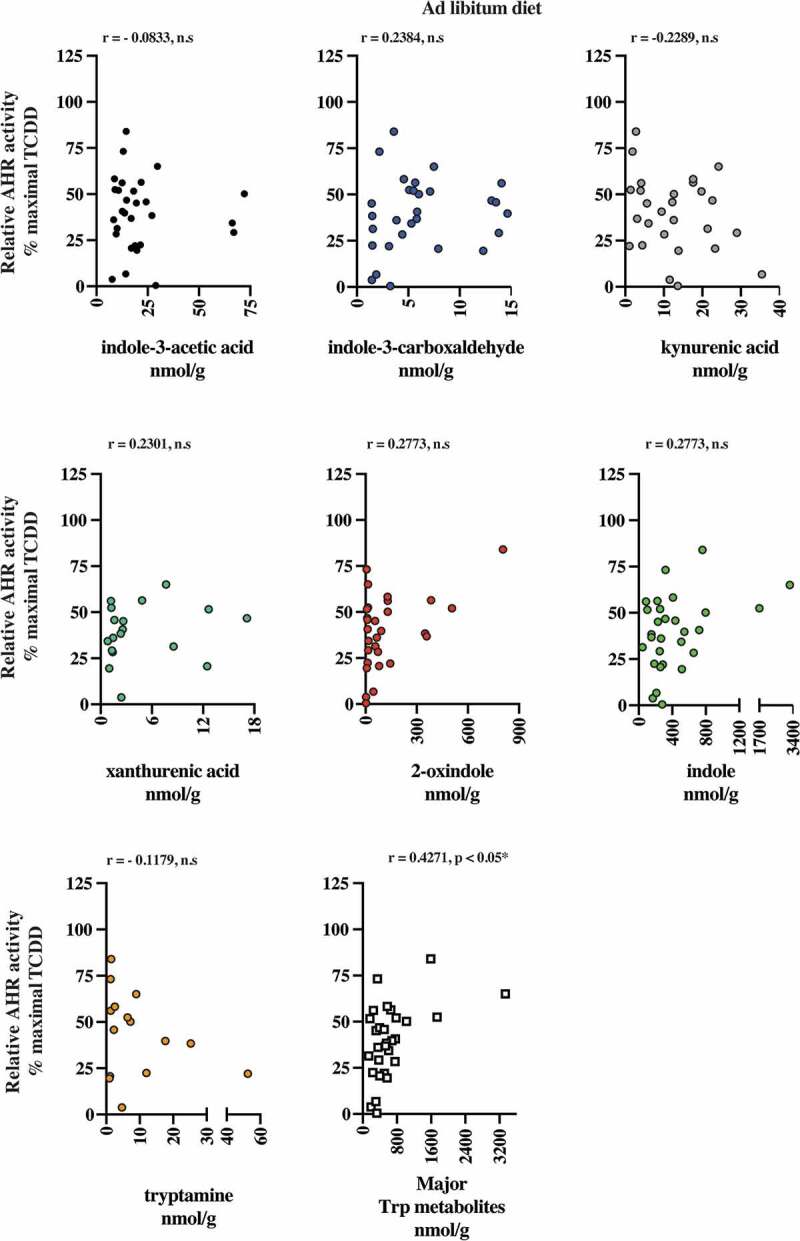
The major tryptophan metabolites levels pooled together correlated with induced AHR transcriptional activity in human fecal extracts from the ad libitum diet group. Six tryptophan metabolites that were present in more than 50% of the fecal samples were correlated individually with AHR transcriptional activity induced HepG2 40/6 cell by fecal extracts. Also, the six metabolites pooled together were correlated with AHR transcriptional activity. Each graph contains the Pearson *r* value correlation coefficient and two-tailed *p* value.

The presence of AHR active, largely microbiota-dependent tryptophan metabolites within human stool prompted us to examine whether a relationship exists between AHR activity and the total pool of extractable metabolites. Extracts of human fecal matter obtained from the defined feeding study participants and those with unrestricted ad libitum diets were examined for their capacity to stimulate activity in a human AHR-dependent luciferase reporter assay system (Figure 7). Following a 5 h exposure in HepG2 40/6 cells, extracts (200-fold dilution) from both dietary cohorts demonstrated a similar level of variation in luciferase reporter output, when expressed as a percentage of the maximal TCDD response. Next, we examined by correlation analyses, whether any relationships exist between individual tryptophan metabolite abundance in both defined diet and ad libitum fecal extracts and AHR activity (Table 6 and Figure 8–9). For the defined diet cohort, significant positive correlations were observed between HepG2 40/6 AHR-dependent reporter activity and the abundance of 2-oxindole and indole-3-carboxaldehyde. A significant positive correlation was also observed between human AHR reporter activity and the cumulative abundance of the indicated tryptophan metabolites. Similar analyses performed with the ad libitum fecal cohort failed to identify any significant correlations between individual tryptophan metabolites and human AHR activity (Table 6 and Figure 9). In these samples, however, a significant positive correlation was observed between the cumulative abundance of the indicated metabolites and human AHR activity.

**Table 6.** Spearman rank correlation between metabolite abundance and AHR activity. Quantifiable tryptophan metabolite abundance represented in >50% of human fecal samples obtained from subjects on a defined or *ad libitum* diet were correlated against AHR-dependent reporter activity in human HepG2 (40/6) cells through non-parametric Spearman rank analysis. Trp metabolites represent the combined abundance of indicated individual metabolites.

Defined diet human fecal metabolite v AHR activity			*Ad libitum* diet human fecal metabolite v AHR activity		
Compound	Spearman *r*	Significance *p* (two-tailed)	Compound	Spearman *r*	Significance *p* (two-tailed)
Indole-3- acetic acid	−0.0997	n.s.	Indole-3- acetic acid	−0.0833	n.s.
Indole-3-carboxyaldehyde	0.4854	< 0.05, *	Indole-3-carboxyaldehyde	0.2384	n.s.
Kynurenic acid	0.0712	n.s.	Kynurenic acid	−0.2289	n.s.
Xanthurenic acid	0.1978	n.s.	Xanthurenic acid	0.2301	n.s.
2-oxindole	0.6191	< 0.0001, ****	2-oxindole	0.2911	n.s.
Indole	−0.0071	n.s.	Indole	0.2773	n.s.
			Tryptamine	−0.1179	n.s.
Trp metabolites	0.5718	< 0.0001, ****	Trp metabolites	0.4271	0.0208, *

### Modulation of microbiota-dependent cecal tryptophan metabolites by diet

**Figure 10.**
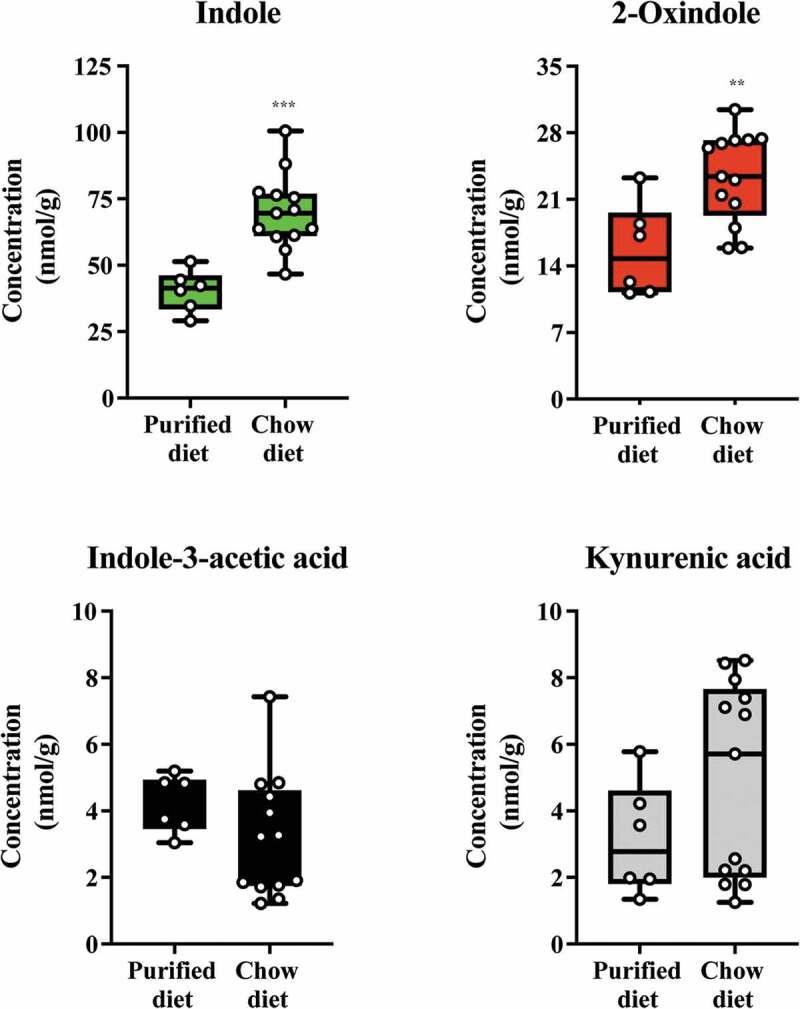
Diet alters levels of indole and 2-oxindole in cecal contents. Mice were fed either a chow or switched to a semi-purified AIN 93 G diet for one week. The levels of indole, 2-oxindole, indole-3-acetic acid and kynurenic acid were determined. Data groups were compared with a Student test. Each box represents the median value with *Q*1 and *Q*3 range, whiskers describe the minimal and maximal values. ** *P* < .01, *** *P* < .001.

Having validated the relative AHR activation potential of the major tryptophan metabolites detectable in mouse cecal contents, we investigated whether their abundance could be modulated through short-term dietary change (Figure 10). Mice with ad libitum access to standard rodent chow diet were switched onto a semi-purified diet or further maintained on standard chow. After one week, cecal contents were isolated and the major tryptophan metabolites (indole, 2-oxindole, indole-3-acetic acid and kynurenic acid) quantified, as appropriate, through GC/MS and LC/MS. Data obtained revealed significant reductions in the abundance of both indole and 2-oxindole in the cecal contents from the semi-purified cohort when compared to those on standard rodent chow. The abundance of indole-3-acetic acid and kynurenic acid was not significantly different between the two dietary groups. These data suggest that dietary factors can influence the abundance of major tryptophan metabolites.

## Discussion

Studies utilizing genetic modification of AHR signaling or experimental in vivo administration of various AHR ligands, including environmental contaminants, diet-derived, endogenous and pseudo-endogenous microbiota-derived compounds, clearly implicate the AHR as a regulator of multiple aspects of gastrointestinal function.^45,46^ However, the majority of these studies have examined the role of AHR ligands in the context of gastrointestinal challenges, disease models or toxicological endpoints. Here we have identified and quantified a panel of predominantly microbiota-derived tryptophan metabolites detectable in mouse cecal contents and human stool that exhibit varying degrees of AHR activation potential. Importantly, these data indicate that intestinal AHR activation is likely to occur at the cecal/fecal concentrations detected. The concentrations of tryptophan metabolites reported here represent cecal/fecal levels rather than enterocyte or serum concentrations and thus may be sequestered or unavailable with regards to intestinal AHR activation. Previous studies however have quantified serum levels of microbiota-derived tryptophan metabolites, e.g. indole and are thus indicative of transepithelial absorption. Additionally, following direct intra-cecal administration of indole to rats, a spike in extraintestinal indole is observed.^47^ Furthermore, studies comparing the serum metabolome of conventionally housed and germfree rodents reported that 10% of identifiable serum features, comprising both microbial and host-microbe co-metabolites, varied by >50% suggesting that, despite its dominant role as a physical barrier, the gastrointestinal epithelium is permissive for microbial metabolite absorption.^48^ Importantly, the serum concentrations of indole and other microbially derived tryptophan metabolites exceed that required to induce AHR activity in cell culture and thus may contribute, under normative conditions, to physiological AHR activities within the gastrointestinal tract.

For mammals, tryptophan represents the least abundant essential amino acid required for protein synthesis. Circulating free tryptophan levels are tightly maintained through binding to albumin,^49^ and metabolism, primarily through degradation by the kynurenine pathway through the activities of tryptophan and indolamine dioxygenases (TDO/IDO1/IDO2).^50,51^ A number of interdependent tryptophan sources for the AHR-sensitive metabolites are suggested. Proteolytic digestion of dietary protein in the small intestine is the primary source of tryptophan. The small intestine, however, is also the major site of tryptophan (and other amino acid) absorption.^52^ Microbial tryptophan metabolism in the small intestine likely provides an in situ source of AHR-sensitive metabolites and activity; in fact, ileal lactobacilli-mediated tryptophan catabolism has already been demonstrated in mice.^12^ Thus, the abundance of tryptophan metabolites detected in cecal and fecal samples suggests either sufficient dietary tryptophan escapes absorption or originates in the small intestine and transits along the gastrointestinal tract,^53^ or a source of tryptophan exists in the large intestine. It has been suggested that tryptophan recycled from exfoliated intestinal epithelial cells and dead bacteria may provide a tryptophan reservoir in the large intestine. Additionally, the high levels of microbiota-derived indole within the large intestine may facilitate retrograde tryptophan synthesis from indole and thus generate a source for in-situ generation of AHR-sensitive tryptophan metabolites.

Notwithstanding the origin of tryptophan, it is clear that multiple tryptophan metabolites are generated within the gastrointestinal tract. To the best of our knowledge, this study provides the first quantitative assessment of a panel of tryptophan metabolites, in mouse cecal contents and human feces, and their relevance to AHR activation potential. A key feature of the current study is the differing concentrations of the various metabolites. Consistent with previous reports, it is clear that indole, in both mouse cecal contents and human stool, represents the most abundant of the tryptophan metabolites examined.^54^ Another key feature of this study is the prominent species-dependent activity of these microbial tryptophan metabolites with regard to either mouse or human AHR. Previously, we have demonstrated that bacterial tryptophan metabolites (e.g. indole, 2-oxindole, 3-methyl indole, kynurenic acid, 2,8-dihydroquinoline) manifest as AHR activators with marked species specificity, exhibiting greater efficacy with the human compared to mouse AHR.^20,32,55,56^ Indeed, at the concentrations detected here, indole can activate mouse AHR. With human AHR however, we had to perform a 20-fold dilution of the concentration quantified in human stool to reduce AHR activity to below saturation. One potential concern in the current and previously published studies is that the species comparisons for a given tryptophan metabolite were performed in two different cell lines and the results could be influenced by metabolism or uptake of the AHR activator. However, we have published a study utilizing primary mouse hepatocytes from either C57BL6/J or a transgenic line, B6. Cg-Ahr^tm1Bra^ Tg (Ttr- AHR)1Ghp that only expresses human AHR in hepatocytes, which allows the testing of ligand activation potential within the same cell type. The tryptophan metabolite indirubin exhibited ~100-fold greater activation potential in the humanized mouse hepatocytes relative to non-humanized cells.^57^ Such species specificity with regard to tryptophan metabolites (and conversely with environmental pollutant AHR ligands) appears to be a common feature, which has been acquired during hominin evolution.^58^ These data would suggest that these major metabolites dominate with regard to dictating AHR activity, this is particularly evident in humans and may question the relevance of rodent studies being used to investigate microbial metabolites and AHR activity. Our studies utilizing GF mice support previous studies indicating that many of the tryptophan metabolites examined are either entirely or in part dependent upon the microbiota.^59^ Such an observation clearly suggests that microbial activity represents a significant source of gastrointestinal AHR activity.

The high concentrations and prevalence of these metabolites would suggest that their microbial synthesis is not restricted to a single species but rather represent common routes of tryptophan metabolism across many species. As such, temporal fluctuations within the community structure of the microbiota may not be evident with regard to the tryptophan metabolite signature. Quantification of cecal indole and 2-oxindole levels from mice on semi-purified or standard rodent chow, that likely having differing microbiota, appears to contradict this conclusion. These data suggest that diets deficient in plant constituents either alter the microbiota structure or its capacity to catabolize tryptophan and generate AHR activators. It remains to be established if the observed reduction in indole and 2-oxindole upon exposure to a semi-purified diet has any influence on AHR activity. Similarly, our data, quantifying the individual and cumulative abundance of the major metabolites in human stool derived from two dietary cohorts (again with presumably different microbial communities) would similarly appear to contradict this notion. Longitudinal fecal metabolite/microbiota analyses in the same individual over time may address this question. In contrast, the minor tryptophan metabolites, such as 2, 8-dihydroxyquinoline, may be restricted to a select subset of the microbiota and as such fluctuation within the microbiota community may have a relatively greater impact on their abundance. Our detection of tryptophan metabolites was restricted to cecal or fecal samples; it remains to be determined if the levels of these metabolites demonstrate any spatial segregation along the gastrointestinal tract in accordance with specific microbiota niches. Future analyses combined with metagenomics may reveal locus-microbiota-metabolite niches of AHR activity in the gastrointestinal tract.

The fact that indole activates the AHR and is highly abundant in stool suggests that it may, in the absence of confounding high-affinity xenobiotic or dietary ligands, the dominant source of AHR activation in the gastrointestinal tract. However, correlation analyses between indole levels and AHR activity appear to contradict this notion. A positive correlation was observed between 2-oxindole and AHR activity in the defined feeding but not with the ad libitum cohort. While indole failed to exhibit a significant relationship with overall fecal AHR activity, this may be due to variable levels of the other major indoles in each individual sample. Additionally, the discrepancy between these observations may involve the presence of, as yet uncharacterized, metabolites of relatively low abundance but high affinity for AHR. Further, more technically challenging mass spectrometry studies may be required to identify such metabolites. Additionally, we cannot discount the possibility that the presence of unknown inhibitors of AHR activity is similarly present in fecal extracts. The potential variable abundance of inhibitors would likely confound any correlative analyses. Yet another potential mechanism that can modulate AHR activation is the presence of CYP1A1 inhibitors/competitive substrates that can increase the half-life of AHR ligands, such as FICZ or serotonin.^10,44,60^ With the exception of 2-oxindole, no single tryptophan metabolite could account for the observed AHR activity across both cohorts. Previous metabolomic analyses examining serum concentrations of microbially derived metabolites are indicative of gastrointestinal absorption; however, to our knowledge, the active transport or passive absorption kinetics of the tryptophan metabolites studied here have not been elucidated in vivo. Our quantification of cecal/fecal tryptophan metabolites represents the maximal available concentration within each sample and fails to account for selective rates of absorption across the individual metabolites. Similarly, the post-absorptive disposition and metabolism of each metabolite are likely to be variable thus confounding the correlative analysis between individual metabolites and AHR activity. Nevertheless, the cumulative abundance of major tryptophan metabolites, however, provided a robustly significant correlation with AHR activity. Thus, we speculate that the compilation of the major metabolites is cooperatively responsible for increased AHR activity. Based upon this observation, we may speculate that fecal screening of these major tryptophan metabolites may represent a useful tool to assess AHR activity within the gastrointestinal tract and may be associated with various pathologies.

Clearly, the microbiota is not altruistic in the generation of host AHR-sensitive tryptophan metabolites. Indole and indole-based compounds exhibit microbial quorum-sensing activity.^61,62^ It is also probable that individual tryptophan metabolites serve as interbacterial cross-feeding intermediates that facilitate particular community niches. The detection of some microbially derived tryptophan metabolites in extraintestinal compartments of the host, e.g. indole, indole-3-acetic acid, indole-3-propionic acid in urine and serum, suggests that these metabolites are released by the microbiota and absorbed by the host.^62,63^ It remains to be established whether the novel AHR activator identified from cecal contents/stool, e.g. 5-hydroxyindole-3-acetic acid, is bioavailable in vivo or represent intracellular bacterial tryptophan degradation intermediates sequestered away from the host. Additionally, we cannot discount the likelihood that many of these metabolites exert AHR-independent activity upon the host. Until recently, the AHR was largely considered a xenobiotic sensor and regulator of detoxification. The capacity of AHR to respond to pseudo-endogenous microbial metabolites, at the detected abundance, implies a physiological significance and suggests the AHR acts a sensor of microbial abundance/structure/metabolic activity. The now established and emerging roles of AHR in modulating immune function and gastrointestinal barrier integrity, respectively, suggest that AHR senses and contributes toward gastrointestinal adaptation in the context of a dynamic microbiota in addition to dietary fluctuations and exposure to potential toxicants. However, it appears that a mutualistic environment regarding microbiota-derived tryptophan metabolites and AHR has evolved in order to maintain interkingdom gastrointestinal homeostasis.

It is now evident that the epithelial barrier, and consequently organism-level, homeostasis is profoundly dependent upon interkingdom communication between the host and its associated microbiota. Here, we propose that in the gastrointestinal tract, components of the microbial metabolite signature, specifically multiple tryptophan metabolites, are recognized by AHR at physiologically quantifiable concentrations. Such recognition likely facilitates host AHR-dependent signaling which in turn contributes toward numerous facets of gastrointestinal function, including immunological surveillance and modulation of microbiota to sustain commensalism. Significantly, we observed a marked species difference with regard to the capacity of cecal or fecal tryptophan metabolites to stimulate mouse or human AHR activity under cell culture conditions. Consistently, human AHR exhibited greater sensitivity to these metabolites when compared to mouse AHR. Such observations bring in to question the relevance of mouse studies when examining the role of microbiota-derived ligands and AHR signaling and extrapolating to human gastrointestinal health.

Given the strong relationship between diet, microbiota and the microbial metabolome signature, we hypothesize that dietary modifications leading to an enhanced gastrointestinal tryptophan metabolite profile are likely to increase AHR transcriptional activity. Indeed, livestock studies involving dietary tryptophan supplementation have demonstrated increases in large intestine *CYP1A1* expression associated with enhanced indole and indole-3-acetic acid levels and combined with improved epithelial barrier integrity.^64^ Such dietary modification may provide a therapeutic advantage to restore homeostasis following gastrointestinal insults arising from microbial dysbiosis or disease. Conversely, diets or exposure to antibiotic agents that reduce microbial diversity and restrict bacterial tryptophan metabolism are likely to reduce AHR activity perhaps with adverse consequences with regard to gastrointestinal homeostasis.

## Supplementary Material

Supplemental Material
