# Supplementary material for: Intestinal microbiota-derived tryptophan metabolites are predictive of Ah receptor activity
Source: Gut Microbes. 2020 Aug 12;12(1):1788899. doi: 10.1080/19490976.2020.1788899 (PMC7524359; doi:10.1080/19490976.2020.1788899)
Supplement: Supplemental Material [file KGMI_A_1788899_SM3941.pdf]

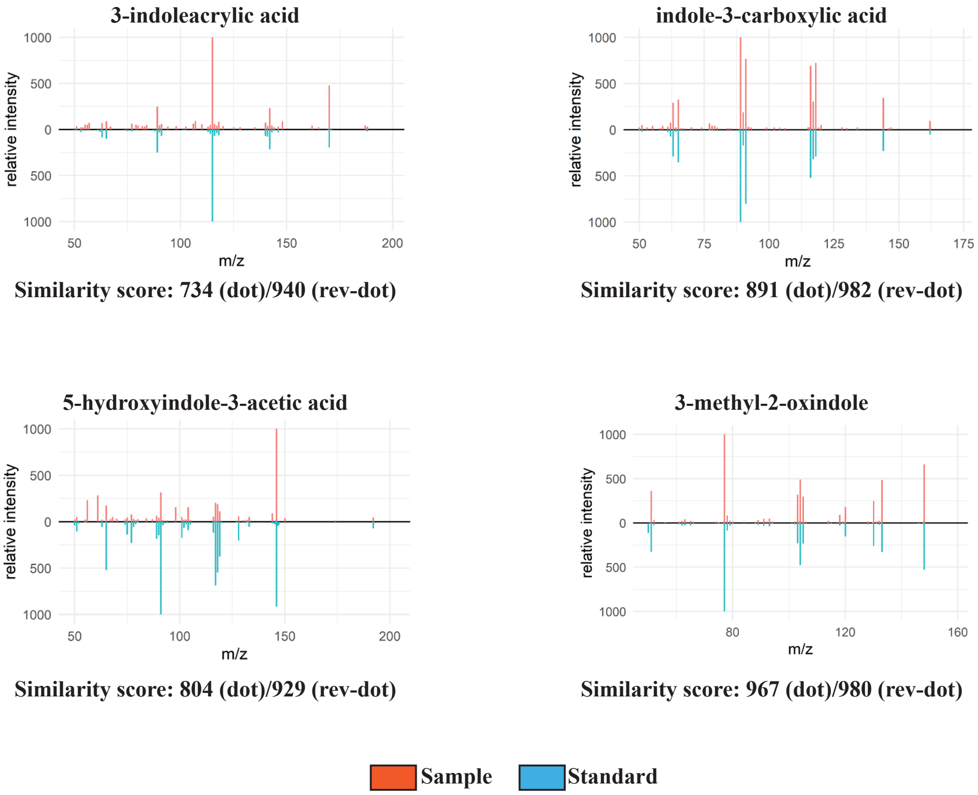

Figure S1. LC/MS/MS scoring validation of identity between standards and indicated tryptophan metabolites which exhibit novel AHR-dependent activity and detected in mouse cecal contents or human feces.

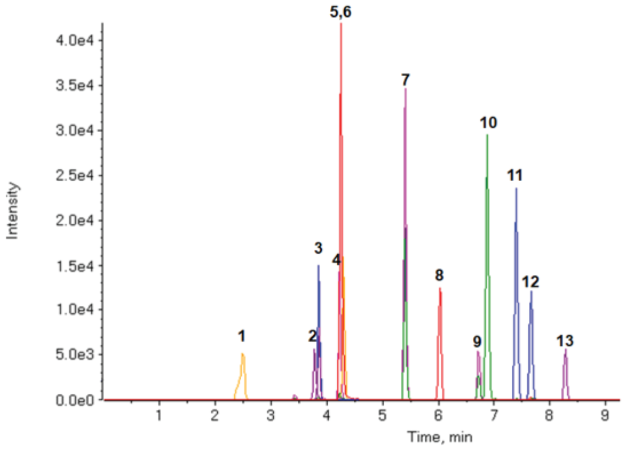

Compound	RT (min)
1. Serotonin	2.50
2. Xanthurenic acid	3.78
3. Tryptophan	3.85
4. 5-Hydroxyindole-3-acetic acid	4.23
5. Kynurenic acid	4.25
6. Tryptamine	4.29
7. 2,8-Dihydroxyquinoline	5.40
8. 2-Oxindole	6.03
9. Indole-3-carboxylic acid	6.72
10. Indole-3-carboxaldehyde	6.87
11. Indole-3-acetic acid	7.39
12. 3-Methyl-2-oxindole	7.66
13. 3-Indoleacrylic acid	8.27

Figure S2. Representative UHPLC chromatogram of indicated daughter ion retention times (Table S1) associated with the tryptophan metabolites

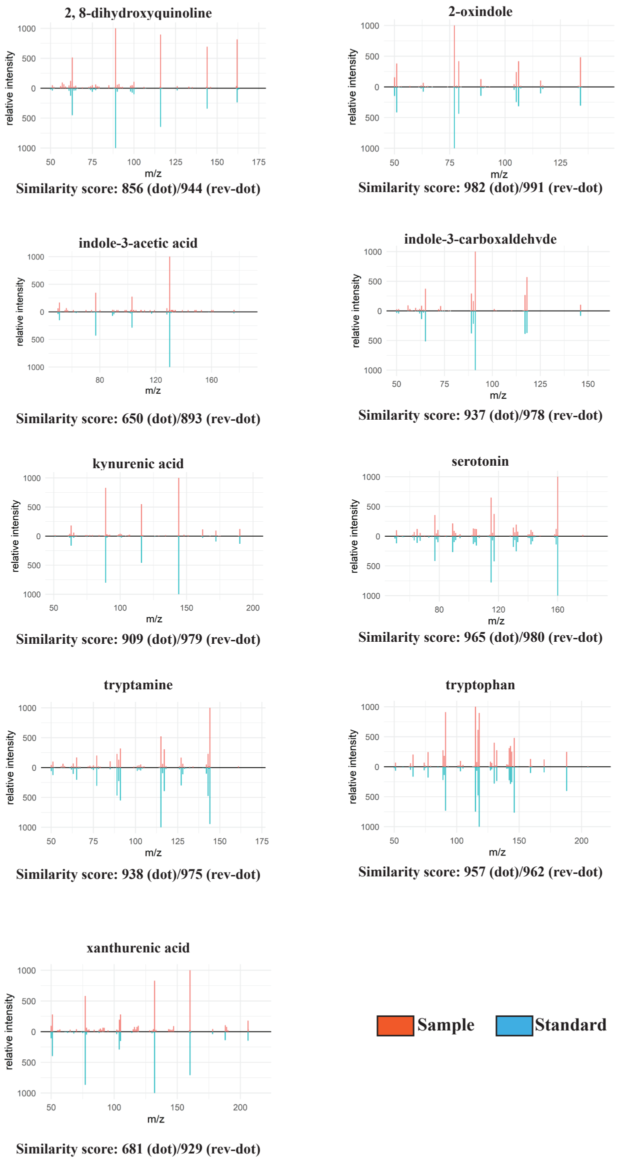

Figure S3. LC/MS/MS scoring validation of identity between standards and indicated tryptophan metabolites detected in mouse cecal contents or human feces.

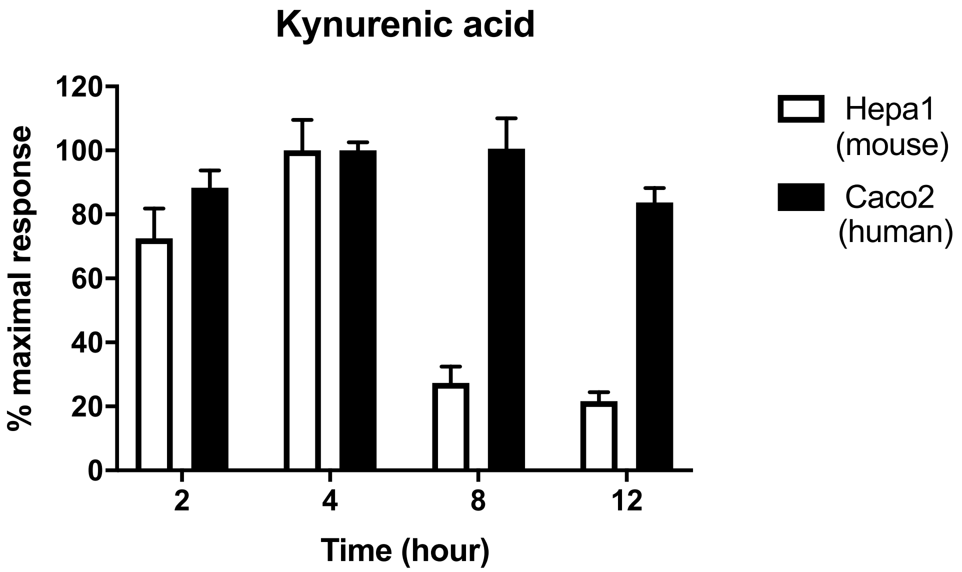

Figure S4. Time course assessment of Cyp1a1 mRNA induction after kynurenic acid treatment of Caco2 and Hepa 1 cells.

Table S1. List of cecal/fecal tryptophan metabolites quantified by a combination of LC/MS and GC/MS platforms and their extracted ions/fragments.

Compound	Chemical composition	Parent ion	Daughter ion/fragments	Platform
Indole-3-acetic acid	C10H9NO2	175.06	130.06	LC/MS/MS
3-Indoleacrylic acid	C11H9NO2	187.06	115.02	LC/MS/MS
Tryptamine	C10H12N2	160.10	144.08	LC/MS/MS
Serotonin	C10H12N2O	176.09	160.05	LC/MS/MS
Indole-3-carboxaldehyde	C9H7NO	145.05	91.05	LC/MS/MS
2,8-Dihydroxyquinoline	C9H7NO2	161.05	116.02	LC/MS/MS
Kynurenic acid	C10H7NO3	189.04	144.03	LC/MS/MS
Xanthurenic acid	C10H7NO4	205.04	132.01	LC/MS/MS
2-Oxindole	C8H7NO	133.05	77.01	LC/MS/MS
Indole-3-carboxylic acid	C9H7NO2	161.05	89.01	LC/MS/MS
3-Methyl-2-oxindole	C9H9NO	147.07	104.03	LC/MS/MS
5-Hydroxyindole-3-acetic acid	C10H9NO3	191.06	146.03	LC/MS/MS
Tryptophan	C11H12N2O2	204.09	118.07	LC/MS/MS
Indole	C8H7N	/	117, 90, 63, 50, 74	GC/MS
3-Methylindole	C9H9N	/	130, 131, 77, 51, 103	GC/MS

Table S2. Range of linearity and correlation coefficient (R^2) for the quantitation of AHR-related compounds.

**Compound**	**Linear equation**	**R square**	**Linearity range (nM)**
Indole-3-acetic acid	y = 0.0004x + 0.0146	0.9918	10-1000
3-Indoleacrylic acid	y = 0.0002x + 0.0015	0.9991	10-1000
Tryptamine	y = 0.0002x + 0.0090	0.9880	10-1000
Serotonin	y = 0.00007x - 0.0014	0.9824	10-1000
Indole-3-carboxaldehyde	y = 0.0005x + 0.0192	0.9930	10-1000
2,8-Dihydroxyquinoline	y = 0.0005x + 0.0198	0.9860	30-2000
Kynurenic acid	y = 0.0008x + 0.0322	0.9903	10-1000
Xanthurenic acid	y = 0.0006x + 0.0040	0.9990	10-1000
2-Oxindole	y = 0.0003x + 0.0043	0.9978	10-1000
Indole-3-carboxylic acid	y = 0.0001x + 0.0011	0.9990	10-1000
3-Methyl-2-oxindole	y = 0.0003x + 0.0042	0.9989	10-1000
5-Hydroxyindole-3-acetic acid	y = 0.0002x + 0.0036	0.9915	10-1000
Tryptophan	y = 0.0001x + 0.0979	0.9805	100-10000
Indole	y = 0.0081x - 0.0032	0.9986	2000-100000
3-Methylindole	y = 0.0106x - 0.0792	0.9908	2000-100000

Table S3. Parameters of Trp metabolite quantification in cecal contents of pathogen-free mice.[a]

Compound	LOQ (nmol/g)	Concentration (nmol/g)	Recovery (%)	Matrix effect (%)
Indole-3-acetic acid	0.07	4.57 ± 0.50	130.2 ± 14.4	58.2 ± 6.3
3-Indoleacrylic acid	0.06	0.09 ± 0.02	147.6 ± 43.6	100.7 ± 14.4
Tryptamine	0.05	3.62 ± 0.62	87.6 ± 1.1	79.0 ± 1.9
Serotonin	0.33	0.85 ± 0.13	139.8 ± 28.1	62.2 ± 8.0
Indole-3-carboxaldehyde	0.06	4.74 ± 0.53	119.2 ± 8.5	81.6 ± 1.6
2,8-Dihydroxyquinoline	0.26	4.63 ± 1.34	116.6 ± 11.8	69.3 ± 3.0
Kynurenic acid	0.16	7.43 ± 0.34	94.6 ± 6.2	87.1 ± 2.9
Xanthurenic acid	0.18	0.66 ± 0.04	106.5 ± 8.9	95.5 ± 6.7
2-Oxindole	0.08	26.28 ± 1.03	99.3 ± 1.9	74.0 ± 0.7
Indole-3-carboxylic acid	0.33	0.62 ± 0.09	122.1 ± 20.6	62.2 ± 8.4
5-Hydroxyindole-3-acetic acid	0.38	0.84 ± 0.17	81.2 ± 9.5	62.4 ± 6.1
Indole	2.00	71.29 ± 4.58	94.9 ± 1.9	94.4 ± 4.4

[a]: Both 3-methyl indole and 3-methyl-2-oxindole were below LOQ.

Table S4. Parameters of Trp metabolite quantification in defined diet human feces.[a]

Compound	LOQ (nmol/g)	Number of detected samples N= 44[b]	Concentration (nmol/g)[c]	Recovery (%)	Matrix effect (%)
indole-3-acetic acid	0.20	44	9.90±12.17	37.2	59.6±6.4
3-indoleacrylic acid	0.22	4	0.48±0.05	35.4	24.3±11.2
tryptamine	0.09	12	5.61±6.21	72.3	83.2±13.3
serotonin	0.13	4	5.92±8.65	43.7	61.31±32.5
indole-3-carboxaldehyde	0.04	44	2.48±2.22	49.7	121.6±38.5
2,8-dihydroxy-quinoline	0.02	19	0.86±0.87	21.5	97.7±32.6
kynurenic acid	1.41	44	3.77±2.70	36.5	106.4±28.0
xanthurenic acid	0.21	30	0.96±0.63	30.3	135.7±43.1
2-oxindole	0.04	41	37.93±45.91	27.7	86.5±11.6
3-methyl-2-oxindole	0.03	15	3.45±5.59	34.4	44.7±22.5
5-hydroindole-3-acetic acid	0.85	21	0.69±0.26	58.0	60.8±14.0
indole	24.27	44	192.53±212.33	93.0	104.0±14.7
3-methylindole	22.61	17	84.0±62.03	102.3	302.8±122.6

[a]: Indole-3-carboxylic acid was below LOQ.
[b]: Indicates the total number of samples.
[c]: Concentration of each compound represents mean +/- SEM concentration across only those samples quantified as >LOQ.

Table S5. Parameters of Trp metabolite quantification in Ad libitum diet human feces.[a]

**Compound**	**LOQ (nmol/g)**	**Number of detected samples n=29[b]**	**Observed concentration (nmol/g)[c]**	**Recovery (%)**	**Matrix effect (%)**
indole-3-acetic acid	0.37	29	4.80±3.83	37.2	59.6±6.4
tryptamine	0.12	15	5.84±8.28	72.3	83.2±13.3
serotonin	0.09	7	3.09±1.67	43.7	61.3±32.5
indole-3-carboxaldehyde	0.03	29	3.69±2.60	49.7	121.6±38.5
2,8-dihydroxy-quinoline	0.17	8	0.83±0.37	21.5	97.7±32.6
kynurenic acid	0.28	27	4.90±3.57	36.5	106.4±28.0
xanthurenic acid	0.13	18	1.90±2.03	30.3	135.7±43.1
2-oxindole	0.07	29	29.19±44.76	27.7	86.5±11.6
indole-3-carboxylic acid	0.17	3	1.74±0.62	25.5	93.5±12.5
3-methyl-2-oxindole	0.01	8	2.41±2.92	34.4	44.7±22.5
5-hydroxyindole-3-acetic acid	0.16	14	0.72±0.31	58.0	60.8±14.0
indole	8.09	29	469.75±601.96	93.0	104.0±14.7
3-methlyindole	7.53	6	229.84±243.62	102.3	302.8±122.6

[a]:3-indoleacrylic acid was below LOQ.
[b]: Indicates the total number of samples.
[c]: Concentration of each compound represents mean +/- SEM concentration across only those samples quantified as >LOQ.
